# Antiglycation and antitumoral activity of *Tribulus terrestris* dry extract

**Published:** 2021

**Authors:** Célia Cristina Malaguti Figueiredo, Amanda Costa Gomes, Filipe Oliveira Granero, João Luiz Bronzel Junior, Luciana Pereira Silva, Ana Lúcia Tasca Gois Ruiz, Regildo Márcio Gonçalves da Silva

**Affiliations:** 1 *São Paulo State University (UNESP), Institute of Chemistry, Araraquara, São Paulo, Brazil*; 2 *Fundação Educacional do Município de Assis (FEMA), Assis, São Paulo, Brazil*; 3 *University of Campinas (UNICAMP), Faculty of Pharmaceutical Sciences, Campinas, São Paulo, Brazil*; 4 *São Paulo State University (UNESP), School of Sciences, Humanities and Languages, Department of Biotechnology, Laboratory of Herbal Medicine and Natural Products, Assis, São Paulo, Brazil*

**Keywords:** Tribulus terrestris, Antiproliferative, Protein glycation, Steroidal saponins

## Abstract

**Objective:**

Investigation of the antiglycation and antitumoral potential of standardized and saponins-enriched extracts of *Tribulus terrestris* herbal medicine.

**Materials and Methods:**

The procedures for the evaluation of the antiglycation activity of the standardized (TtSE) and saponins-enriched (TtEE) extracts of *T. terrestris* were: determination of relative mobility in electrophoresis (RME), free amino groups using OPA method and advanced glycation end-products (AGEs) fluorescence. Antioxidant activity was determined by DPPH radical scavenging test. *In vitro* antitumor activity of TtSE and TtEE was evaluated in human tumor cell lines.

**Results:**

The results were obtained by antiglycation tests (RME, OPA method and AGEs fluorescence determination), using BSA as protein and ribose as glycation agent, and antioxidant assay (DPPH test); it was verified that both extracts of *T. terrestris* have antiglycation and antioxidant activity. In addition, the extracts were able to induce death of more than 50% of human tumor cell lines.

**Conclusion:**

The present study showed that standardized and saponins-enriched extracts of *T. terrestris* herbal medicine present antiglycation and antioxidant and antiproliferative action in human tumor cells lines. The saponins-enriched extract proved a greater antiglycation and antioxidant activity in comparison to the standardized type.

## Introduction

Glycation is a process that involves the non-enzymatic addition of reducing sugars and/or their reactive degradation products in free-amino groups of proteins, lipids or nucleic acids that can occur under physiological and pathological conditions (Gugliucci et al., 2009 ▶). This process results in structure and function alterations in these biomolecules in cells and body tissues due to increases in advanced glycation end-products (AGEs). AGEs are a heterogeneous class of molecules that interact with the receptor for AGEs and induce increment of oxidative stress and inflammatory mediators (Kouidrat et al., 2015 ▶). The physiological AGEs accumulation has been implicated in development and progression of different chronic diseases including cardiovascular diseases, chronic renal failure, neurodegenerative diseases, diabetes and cancer (Chhipa et al., 2019 ▶; Zeng et al., 2019 ▶).

Studies indicated that secondary metabolites, such as polyphenols, terpenes and steroidal compounds present in herbal medicine, are able to reduce non-enzymatic glycation (Tian et al., 2019 ▶). Besides, these compounds have been associated with prevention and treatment of different types of cancer (Majidinia et al., 2019 ▶; Sajadimaj et al., 2020 ▶). The study of the use of phytotherapeutics can benefit the treatment of diabetic patients both in glycation control and in cancer development and progression (Chhipa et al., 2019 ▶; Krishna et al., 2020 ▶). *Tribulus terrestris *has been studied for its preventive and therapeutic action in different diseases due to avariety of phytoconstituents such as phenols, terpenes and steroids (saponins) present in its extracts (Majidinia et al., 2019 ▶; Sanagoo et al., 2019 ▶).

*Tribulus terrestris* Linn is a dicotyledonous herb that belongs to the Zygophyllaceae family and is widely grown in Africa, Australia, Asia and Europe (Qureshi et al., 2014 ▶). Chinese traditional medicine uses *T. terrestris* to improve visual acuity and as an anticonvulsant, while Indian herbal medicine takes advantage of its diuretic, tonic and aphrodisiac properties (Chhatreet al., 2014 ▶).* T. terrestris *is commonly ingested in its extract form, which is obtained from leaf and stem parts or from fruits that mainly contain steroidal glycosides (saponins) of the furostanol type (Combarieu et al., 2003 ▶). Studies demonstrated that saponin protodioscin isolated from *T. terrestris*, is one of the most active compounds capable of inducing an increase in testosterone production (Su et al., 2009 ▶; Sivapalan, 2016 ▶).

Several pharmacological studies have revealed that the *T. terrestris *extracts have different effects like cardioprotective, hepatoprotective, antitumoral (Angelova et al., 2013 ▶; El-Shaibany et al., 2015 ▶; Pokrywka et al., 2017 ▶; Sisto and Lisi, 2019 ▶), anti-atherosclerotic (Li et al., 2013 ▶), antiarthritic (Mishra et al., 2013 ▶), antioxidant (Mohd et al., 2012 ▶), antimicrobial (Akbal et al., 2018 ▶), analgesic and anti-inflammatory properties (Oh et al., 2012 ▶),and can be used in treatment of oral infections and control dental caries and periodontal diseases (Soleimanpour et al., 2015 ▶). Studies showed the extract of *T. terrestris* is effective in treating hyperglycemia *in vivo* and inhibiting α‐glucosidase and aldose reductase *in vitro *(Lamba et al., 2011 ▶), and in treatment of pancreatitis in mice (Borran et al., 2017 ▶). Therefore, the present study aimed to investigate the antiglycation and antitumor potential of standardized and saponins-enriched extracts of *T. terrestris*. To the best of our knowledge, there are no data as such on the correlation between the antiglycation and antitumor activities. Previous studies have mainly discussed steroidal compounds (saponins) present in this herbal medicine. 

## Materials and Methods

**Preparation of the standardized extract of** ***Tribulus terrestris*** **(TtSE)**

The dry extract of *T. terrestris *herbal medicine was obtained directly from suppliers of pharmaceutical raw material (FLORIEN, São Paulo, Brazil). The standardized dry extract containing 43.21% of total saponins was acquired, accompanied by the certification of physical and microbiological quality.

**Preparation of the extract enriched in saponins of** ***T. terrestris*** **(TtEE)**

The extract of *T. terrestris *enriched in saponins was obtained according to Dinchev et al. (2008) ▶ and Ren et al. (2015) ▶, where10 g of the TtSE was added to 250 ml chloroform for 1 hr under stirring at room temperature. The obtained chloroform extract was filtered and again extracted into Soxhlet using250 mlof 70% ethanol for 1 hr. The solvent was removed from the extract and the extract was then lyophilized. The total yield of saponins-enriched extract was determined in grams.

**Determination of total saponins**

The total saponin content of different samples was determined by the double extraction gravimetric method described by Ezeabara et al. (2014) ▶ and Koomson et al. (2018) ▶. Here, 5 g of the powdered sample was weighed and mixed with 50 ml of 20% ethanol solution in a flask. The mixture was heated with periodic agitation in a water bath for 90 min at 55ºC; it was then filtered through a filter paper. The residue was extracted using 50 ml of 20% ethanol, and theextracts were combined, then, reduced to about 40 mlat 90ºC, transferred to a separating funnel and finally, 40 ml of diethyl ether was added and shaken vigorously. Partitioning re-extraction was done repeatedly until the aqueous solution showed a clear-color layer. Saponins were extracted using60 mlbutanol. The combined extracts were washed with 5% sodium chloride (NaCl) solution and this solution was evaporated in a pre-weighed evaporation dish, dried at 60ºC in a laboratory oven and re-weighed after cooling in a desiccator. The process was repeated for two times to obtain an average percentage. Saponin content was calculated as a percentage of the original sample. Percentage (%) of saponins = (W_2 _- W_1 _/ Weight of sample) x 100; where: W_1_ = weight of evaporating dish and W_2_ = weight of evaporating dish + sample.

**Determination of saponins by HPLC**

High Performance Liquid Chromatography (HPLC) analysis of saponins was performed on a Kinetex C18 (150 x 2.1 mm, 5 μm) column (Phenomenex Inc., Torrance, CA, USA). The mobile phase was composed of 0.1% v/v formic acid in acetonitrile (B) and 0.1% v/v formic acid in ultrapure water (A). Gradient elution mode was performed from 5-100% B in A during 40 min with a flow rate of 1 ml/min. The column oven temperature was set to 40°C. Samples were monitored by a Pulsed Amperometric Detection (PAD) detector in the range of 200-800 nm. The injection volume was 2.0 μl.

**Antioxidant activity assessed by DPPH radical scavenging test**

Antioxidant activity was measured by the DPPH (2, 2-diphenyl-1-picryl-hydrazyl-hydrate) method described by Rufino et al. (2010) ▶. The experiments were performed in triplicate for statistical purposes. Solutions containing 1 ml of acetate buffer (pH 5.5 and 100 mM), 1.25 mlethanolAnalytical Standard (AS), 250 μl DPPH solution (500 μM), and 50 μl of the samples, were prepared. TtSE and TtEE reacted with DPPH for 30 min in the dark and absorbance wasmeasured in a spectrophotometer (UV-Vis spectrophotometer – Bel Photonics Brazil) at 517 nm. Antioxidant activity was calculated according to the formula: Antioxidant activity (%) = [(Control-Samples)/Control] x 100. Gallic acid was used as positive control at a concentration of 100 μg/ml. TtSE and TtEE were evaluated at concentrations of 250, 500 and 1000 μg/ml.

**Evaluation of antiglycation activity**

**Evaluated Relative mobility in electrophoresis (RME)**

RME was evaluated according to the methodology described by Miura et al. (1994) ▶. Bovine Seric Albumin (BSA) (0.3 mg/ml protein) (Sigma-Aldrich, Brazil) was diluted in 10 mM Phosphate Buffered Saline (PBS), containing ribose (400 mM) (Sigma-Aldrich, Brazil) and aminoguanidine (1 mM) (Sigma-Aldrich, Brazil) at 37°C for 72 hr in the presence or absence of the extracts (0.1 mg/ml). SDS-PAGE was performed using a 5% stacking gel and a 8% separation gel. An aliquot of glycated BSA solution was mixed with an equal volume of sodium dodecylbenzene sulfonate (SDS - Labsynth, Brazil) sample buffer (20 mg/ml SDS, 30% glycine (Sigma-Aldrich, Brazil), 0.25 M Tris-HCl buffer, pH 6.8) and boiled for 3 min. From the mixed solution, 15 μl was applied to the stacking gel, and the separation gel was run in a constant flow of 30 mA, for approximately 3 hr. After the electrophoresis process, the gel was fixed using acetic acid and then stained with Coomassie-Blue (R-250) (Sigma-Aldrich, Brazil). The results were observed in the RME using BSA. Final concentrations of aminoguanidine (AMG) (1 mM) and BSA (0.3 mg/ml protein, without the addition of the extract) were used as positive and negative controls, respectively.

**Determination of free amino groups**

Free amino groups of glycated samples were determined by the ortho-phthaldialdehyde (OPA) (Sigma-Aldrich, Brazil) method (Fayle et al., 2001 ▶). OPA reagent was immediately prepared before use by mixing 25 ml of 0.1 M sodium borate, 2.5 ml of 20% SDS, 100 µl of 2-mercaptoethanol (Sigma-Aldrich, Brazil) and 40 mg OPA (dissolved in 1 ml of methanol) and adjusting the final volume to 50 ml with distilled water. An aliquot of samples containing 50 µg of BSA was adjusted to 1 ml with OPA reagent, incubated for 2 min at room temperature and absorbance was obtained at 340 nm against a blank containing the OPA reagent. Untreated BSA (control) was assumed to have 100% of free amino groups.

**Determination of advanced glycation end-products (AGEs) fluorescence**

The same reaction mixture used for the determination of free amino groups, was incubated at 37°C for 7 days. The formation of AGEs was determined by measuring its fluorescent characteristics using excitation and emission maximum of 360 and 460 nm, respectively. The process was performed in duplicate and the results are expressed by the percentage of inhibition of AGEs formation.

**Evaluated of antitumoral activity**

The antitumor activity of TtSE and TtEE was evaluated against nine human tumor cell lines [glioblastoma (U251), melanoma (UACC-62), breast cancer (MCF7), doxorubicin-resistant high-grade ovarian serous adenocarcinoma (NCI-ADR/RES), renal cell carcinoma (786-0), large cell lung carcinoma (NCI-H460), high-grade ovarian serous adenocarcinoma (OVCAR-03), rectosigmoid adenocarcinoma (HT-29) and chronic myelogenous leukemia (K562)] and one non-tumorigenic human keratinocyte (HaCaT). The tumor cell lines were provided by the Frederick Cancer Research & Development Center, National Cancer Institute, Frederick, MA, USA, while the non-tumor cell line HaCaT (human keratinocyte) was provided by Dr. Ricardo Della Coletta (University of Campinas-UNICAMP, Brazil). Stock cultures were grown in 5 ml of Roswell Park Memorial Institute (RPMI) 1640 medium (Gibco®, USA) supplemented with 5% fetal bovine serum (Gibco®, USA) at 37°C in 5% CO_2_ with a 1% penicillin (1000 U/ml) - streptomycin mixture (1000 mg/ml) (Vitrocell®, Brazil) (complete medium). Stock solutions of TtSE and TtEE were prepared in dimethylsulfoxide (100 mg/ml) followed by serial dilution on complete medium resulting in the final concentrations of 0.25, 2.5, 25 and 250 μg/ ml. Doxorubicin (DOX) (final concentrations of 0.025, 0.25, 2.5 and 25 μg/ ml in complete medium) were used as a positive control. Cells in 96-well plates (100 μl/well, inoculation density: 3.5 to 6 x 10^4^cell/ml) were exposed to the four concentrations of AuTCEP, TCEP, and doxorubicin (100 μl/well) in triplicate, for 48 hr at 37°C and 5% of CO_2_. Before (T0 plate) and after the addition of the samples (T1 plates), the cells were fixed using50% of trichloroacetic acid (50 μl/well), and cell proliferation was determined by spectrophotometric quantification (540 nm) of the cellular protein content using the sulforhodamine B assay. GI_50_ values (concentration that inhibits 50% of cell growth) were determined through sigmoidal regression using Origin software version 8.0 (OriginLab Corporation, USA) (Monks et al., 1991 ▶; Nunes et al., 2017 ▶).

 **Statistical analysis**

The data obtained in antiglycation and antioxidant assays were evaluated by the BioEstat software version 5.0 through Analysis of Variance (ANOVA) followed by *post hoc* Tukey’s test (p≤0.05).

## Results

**Total saponins before and after enrichment**

In determining the total saponins of TtSE, 40% total saponin content was observed and this value was in accordance with the technical report of the herbal medicine sold in pharmacies, which presents an average 43%. The extraction of TtSE was directed to obtain the saponins-enriched extract (TtEE). This extract presented 72.8% of total saponin content, thus showing an increase of 32.8% in comparison to TtSE.

**Saponins content determined by HPLC**

**Figure 1:**
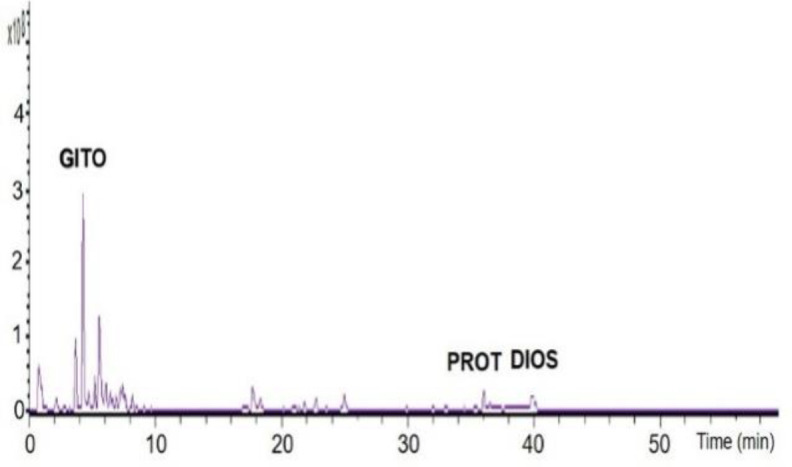
HPLC chromatogram of saponins-enriched extract of T. terrestris herbal medicine. Peak identification: GITO=gitogenin; PROT=protodioscin and DIOS=diosgenin

The method developed for HPLC provided a quick analysis of the herbal medicine extract of *T. terrestris* saponin-enriched. The conditions used led to a good separation of the peaks in standard solution which could be identified in the chromatogram (Figure 1), gitogenin (Rt = 4.9 min.), protodioscin (Rt 37.1 min.) and diosgenin (Rt 37.7 min.), saponins were identified by comparison with the chromatograms as observed in the literature for these species.

**Antioxidant activity determined by DPPH radical scavenging test**

Antioxidant activity assessed by the DPPH test, presented in Table 1, showed that the TtSE presents the highest antioxidant potential at the concentration of 1000 μg/ml (54.45%), while the concentrations of 250 and 500 μg/mlwere not significantly different. The TtEE showed the lowest activity at a concentration of 250 μg/ml (45.97%). However, the greatest antioxidant activities were presented at concentrations of 500 (66.48%) and 1000 μg/ml (70.45%), which did not differ significantly from each other and from the positive control.

**Table 1:** Antioxidant activity of *T. terrestris* standard extract (TtSE), *T. terrestris* saponins-enriched extract (TtEE) and positive control (PC) Gallic acid (100 µg/ml), as assessed by DPPH test

Extract	Concentration (µg ml^-1^)	Antioxidant activity (%)
	250	27.22±2.91a
TtSE	500	36.14±1.04a
	1000	54.45±3.90b
TtEE	250	45.97±4.31b
	500	66.48±3.14c
	1000	70.45±3.17c
PC	100	74.56±0.00c

Mean and standard deviation followed by equal letters in the same column did not differ statistically as examined by the Tukey test (p≤0.05).

**Antiglycation activity of the TtSE and the TtEE**

**Figure 2:**
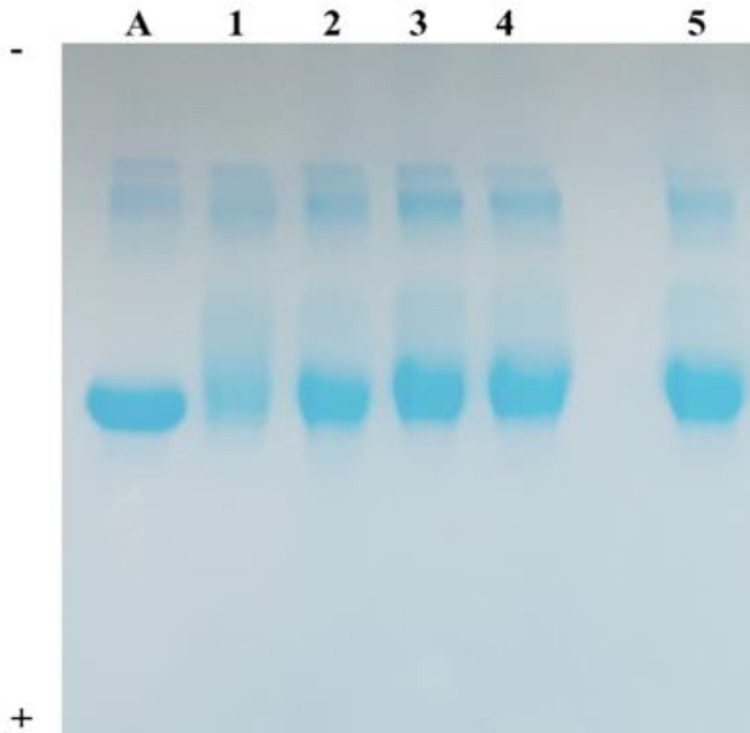
Relative mobility in electrophoresis test for evaluation of antiglycation capacity by system A=BSA; 1=BSA+Ribose; 2=BSA+Ribose+AMG; 3=BSA+Ribose+TtSE; 4=BSA+Ribose+TtEE and 5=BSA+Ribose+Diosgenin. Where BSA=bovine serum albumin, AMG=Aminoguanidine, TtSE=*T. terrestris* standard extract and TtEE=*T. terrestris* saponins-enriched extract

In the antiglycation activity assessment doneby RME analysis, the TtSE and TtEE were evaluated at a concentration of 1000 μg/ml. It is possible to observe in Figure 2 the electrophoretic profiles of BSA (A), treatments with AMG (2), TtSE (3), TtEE (4), and saponin diosgenin (5). It can be observed that the extracts showed antiglycation activity against the glycation promoted by ribose in BSA. BSA treated with TtSE, TtEE and saponin diosgenin exhibited electrophoretic profiles similar to BSA treated with AMG. The glycated BSA with ribose (1) showed an electrophoretic profile of protein degradation.

**Figure 3:**
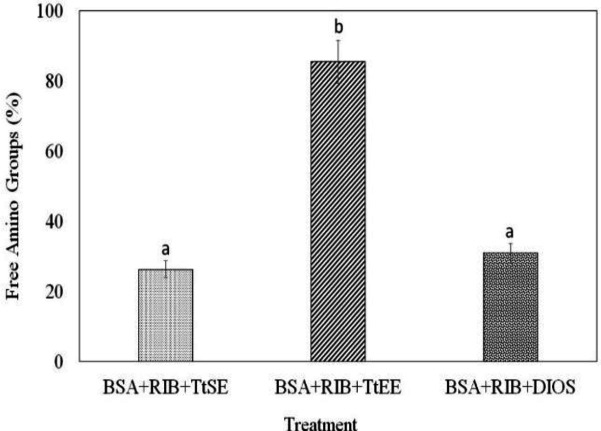
Percentage of free amino groups determined by OPA method with the TtSE (*T. terrestris* standard extract), TtEE (*T. terrestris* saponins-enriched extract) and DIOS (saponin diosgenin) in association with BSA (Bovine Serum Albumin) as protein and RIB (Ribose) as glycation agent. Different letters superscripts indicated mean difference between treatments significant at p<0.05

Figure 3 shows the percentages of free amino groups determined by the OPA method with the TtSE and TtEE in association with BSA as protein and ribose as a glycation agent. It was observed that 45.85% of the free-amino groups are submitted to glycation in the presence of ribose, reflecting the real value of free amino groups in the samples treated with the extracts. Thus, the percentage of free amino groups in the treated samples was 26.33% for TtSE, 85.32% for TtEE and 30.89% for diosgenin.

Moreover, it was possible to observe that BSA treated with TtSE and diosgenin did not show a significant difference in the percentages of free amino groups. The greatest percentage of free amino groups was observed in BSA treated with TtEE (85.32%), differing significantly from the other treatments, and presenting the highest antiglycation activity against ribose.

**Figure 4:**
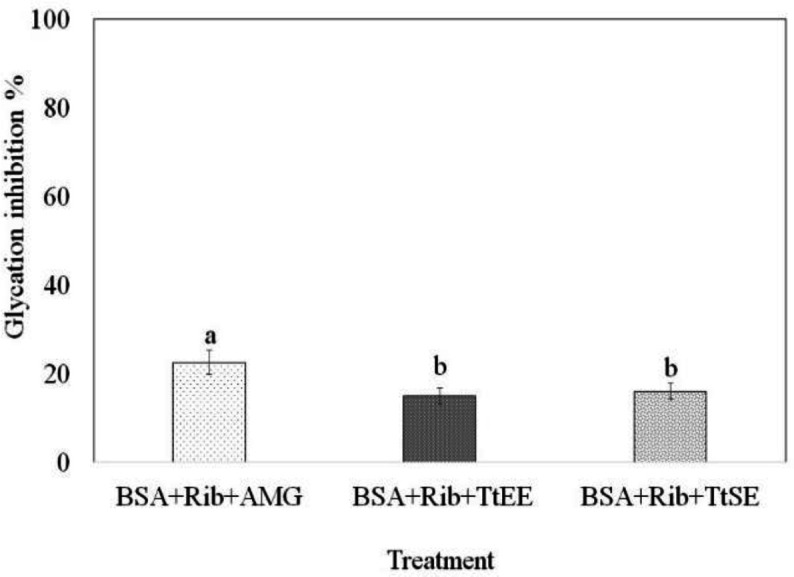
Percentage of glycation inhibition: TtSE (*T. terrestris* standard extract); TrEE (*T. terrestris* saponins-enriched extract); AMG (Aminoguanidine), BSA (Bovine Serum Albumin) and RIB (Ribose).Different letters superscripts indicated mean difference between treatments significant at p<0.05

Figure 4 shows the results of free amino groups determination expressed as the percentage of glycation inhibition; itcan be observed that TtSE and TtEE inhibited glycation by approximately 15% and no significant difference was found between them; however, they differed from the control treated with AMG(22.57%). These results are in accordance with the fluorescence determined for the reaction of the samples evaluated.

**Figure 5:**
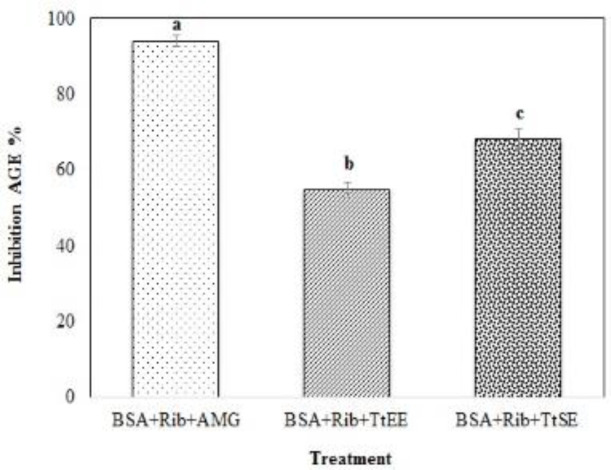
AGEs (Advanced Glycation End-Products) formation inhibition values determined by AGEs fluorescence: TtSE (*T. terrestris* standard extract); TrEE (*T. terrestris* saponins-enriched extract); AMG (Aminoguanidine), BSA (Bovine Serum Albumin) and RIB (Ribose). Different letters superscripts indicated mean difference between treatments significant at p<0.05

 Figure 5 shows the values of inhibition of AGEs formation determined by fluorescence of AGEs excitation and maximum emission at 360 and 460 nm, respectively. It was determined that the two treatments differed significantly from each other and from AMG. However, treatments with TtSE and TtEE inhibited more than 50% of AGEs formation, characterizing the extracts as a source of antiglycation compounds.

**Antitumor activity**

**Figure 6:**
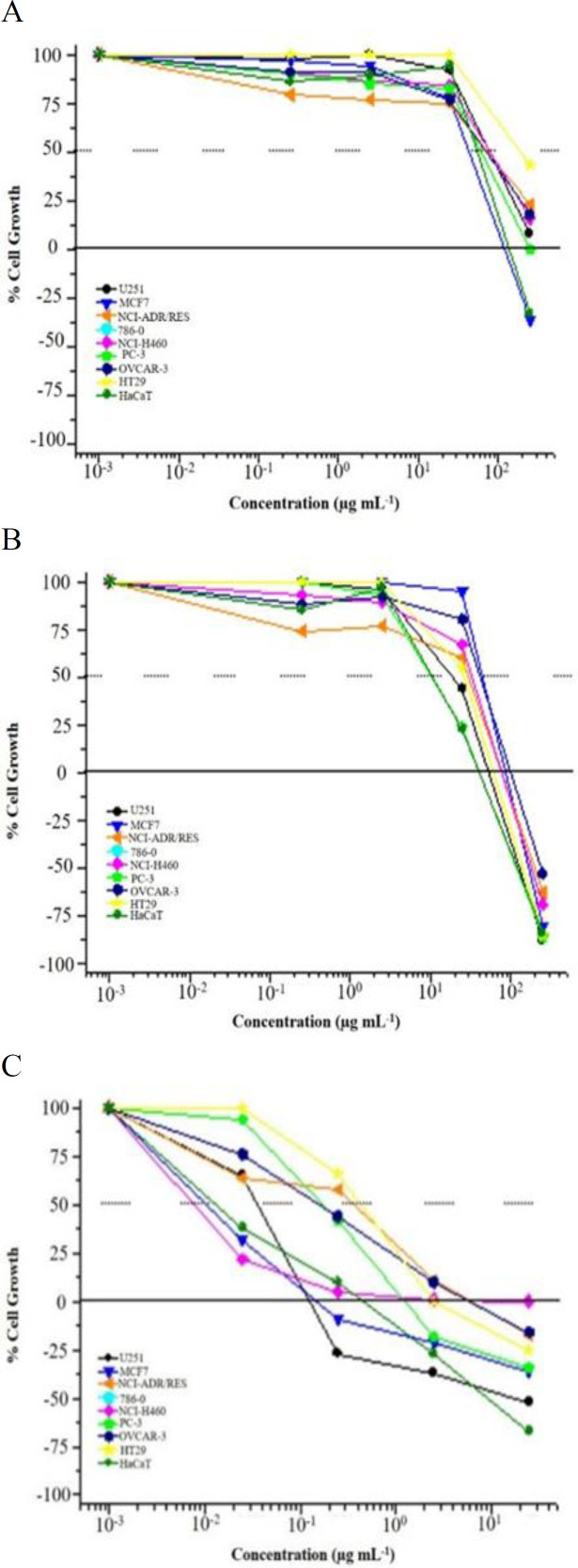
*In vitro *antiproliferative activity of A=TtSE (*T. terrestris* standard extract); B=TrEE (*T. terrestris* saponins-enriched extract) in human tumor cell lines: U25 (glioma), MCF-7 (breast), NCI-ADR/RES (ovary with multidrug resistant phenotypes), 786-O (kidney), NCI-H460 (lung), PC-3 (prostate), OVCAR-03 (ovary), HT-29 (colon) and human non-tumor lineage: q=HaCaT (keratinocyte). Doxorubicin (C) was used as a reference standard

*In vitro *antiproliferative activity of TtSE and TtEE was examined in human tumor cell lines: U25 (glioma), MCF-7 (breast), NCI-ADR / RES (ovary with multidrug-resistant phenotypes), 786-O (kidney), NCI-H460 (lung), PC-3 (prostate), OVCAR-03 (ovary), HT-29 (colon) and human non-tumor lineage: q = HaCaT (keratinocyte). Doxorubicin was used as a reference standard and the results are summarized in Figure 6. 

Results revealed that the TtSE and TtEE exhibited promising antiproliferative activity for tumor cell lines when compared to doxorubicin. Regarding the antiproliferative effect, the TtEE showed higher selectivity for the kidney (786-O) cell line with GI_50_ of 2.91 µg/ml and TtSE presented 30.63 µg/ml and showed moderate activity against the tested cell lines (Table 2).

**Table 2:** Antiproliferative effect of *T. terrestris* standard extract (TtSE) and *T. terrestris* saponins-enriched extract (TtEE) extracts of *T. terrestris *and doxorubicin (DOX) against human cancer cell lines^a^

Cell lines	GI_50_(µg/ml)^b^		
	DOX	TtEE	TtSE
Glioma (U251)	0.026	24.67	79.05
Mama (MCF7)	<0.025	34.24	29.11
Ovary (NCI-ADR-RES)	0.16	26.26	56.01
Kidney (786-O)	<0.025	2.91	30.63
Lung (NCI-H460)	<0.025	27.24	77.40
Prostate (PC-3)	0.23	22.53	43.02
Ovary (OVCAR-3)	0.14	29.49	67.89
Colon (HT-29)	0.26	25.08	243.50
Skin (line of non-tumor cells) (HaCat)	<0.025	21.02	33.17

^a ^Assessed by the SRB assay.

^b ^GI_50_ values represent the concentration (µg/ml) necessary for total inhibition of cancer cells proliferation. Dose range tested: 0.25–250 µg/ml of extracts and 0.025–25 µg/ml of doxorubicin.

## Discussion

Studies conducted on *T. terrestris* and its saponins showed different biological activities against various degenerative diseases mainly promoted by oxidative stress, such as cancer and diabetes (Sadowska-Bartosz and Bartosz, 2016 ▶; Santos et al., 2019 ▶; Jud and Sourij, 2019 ▶). Previous studies using extracts obtained from different parts of the plant (leaves, stem, root and fruits) demonstrated the antitumor effect on the proliferation of different cancer cells (Divya et al., 2014 ▶; Pourali et al., 2017 ▶) and their antiglycation activity was demonstrated in plant screening studies (Siddiqui et al., 2016 ▶). However, studies evaluating antiglycation and antitumor activities of commercial *T. terrestris* herbal medicines are still few and rare in the scientific literature, mainly those correlating these activities with the total steroidal saponins content in herbal medicines.

The enrichment of the *T. terrestris* herbal extract resulted in an increase of 32.8% in the total concentration of saponins, increasing the 40% found in the standard extract to 72.8% in the saponins-enriched extract. Similar results wer ealso demonstrated by Ezeonu and Ejikeme (2016) ▶, the authors quantified the total saponins in the enriched extract of different medicinal plants. Studies carried out by Singh and Chaudhuri (2018) ▶ and Sobolewska et al. (2020) ▶ demonstrated that the total steroidal saponins content present in plant extracts, may be directly correlated with biological activity, both for the prevention and treatment of physiological diffusions and for the toxicity promoted by saponins.

Precision chemical analyses performed on different *T. terrestris* extracts demonstrated the presence of different steroidal saponins classes (furostanol and spirostanol) (Combarieu et al., 2003 ▶; Mulinacci et al., 2003 ▶). In order to verify the variety of saponins in the extract enriched in saponins (TtEE), HPLC/PDA analysis was performed. This analysis demonstrated the presence of gitogenin, protodioscin and diosgenin saponins. However, the peak with the highest intensity was for gitogenin. Similar results were reported by Ivanova et al. (2010) ▶ and Pavin et al. (2018) ▶ using extracts obtained from different parts of *T. terrestris*.

Different authors demonstrated that *T. terrestris* extract has antioxidant potential, mainly as a scavenger of free radicals; this activity was directly correlated with the saponin content found in the extracts (Dinchev et al., 2008 ▶; Hammoda et al., 2013 ▶). In this study, antioxidant activity of both the TtSE and the TtEE extracts was shown by the DPPH test. However, TtEE showed a significantly higher antioxidant activity compared to TtSE. This may be correlated with the presence of greater saponins content for which, the antioxidant activity was demonstrated by Escribano et al. (2017) ▶ and Doost et al. (2019) ▶.

Scientific studies have shown that AGEs generated in glycation processes are able to increase oxidative stress; concomitantly, the increase in free radicals leads to an increase in the glycation activity of sugars in different biological molecules. Therefore, the antioxidant and antiglycation activities are correlated, since these compounds act in the control of oxidative stress and protein glycation (Dil et al., 2019 ▶; Neha et al., 2019 ▶). In the present study, the antiglycation activity of the extracts (TtSE and TtEE) was assessed by*in vitro* bioassays. In the RME test, both extracts showed antiglycation activity against the glycation promoted by ribose on the protein sample (BSA). Similar studies by Gilabert-Oriol et al. (2015) ▶ and Prasad et al. (2019) ▶ demonstrated the antiglycation activity of saponins and other natural compounds on glycating agents.

In the OPA method, antiglycation activity was more prominent in the saponins-enriched extract. Studies performed by Elekofehinti (2015) ▶ using the OPA method for evaluation of antiglycation activity of steroidal saponins associated the antiglycation action with the reduction of complications caused by diseases, such as diabetes mellitus. This benefit probably correlates with the antiglycemic action, as inhibits the glycation of proteins and other biomolecules, as demonstrated by Younus and Anwar (2016) ▶.

In the determination of free amino groups and inhibition of AGEs formation, the results showed antiglycation activity of both extracts; however, the TtEE presented a greater activity compared to the TtSE. These results are reported for the first time on scientific bases, therefore, there are no similar results that can be compared or interpolated to the analyses carried out in the present study. However, the frequency of citations about the antiglycation potential attributed to *T. terrestris* extracts and their isolated compounds may also be correlated with the antioxidant activity (Sousa et al., 2015 ▶; Naz et al., 2017 ▶).

In view of the antiglycation results observed in the present study, it is known that the decrease and even suppression of the physiological production of AGEs can prevent/treat the damage caused by diabetes and even reduce the emergence of different types of cancers related to glycation (García-Jiménez et al., 2016). The AGEs mainly affect long-lived proteins, like hemoglobin, collagen, or elastin (Nadjib et al., 2018 ▶; Bansode and Gacche, 2019 ▶). Effects of glycation and AGEs generation on short-lived proteins, including plasma albumin, are also of interest because they cause several structural and functional modifications (Zendjabil, 2020 ▶). For this reason, study of the impacts of glycation in albumin, is an emerging trend. *In vitro* model systems of protein glycation using BSA (BSA has approximately 76% sequence homology to human serum albumin) incubated with sugars,have appeared as an interesting option to investigate deleterious consequences of protein glycation (Rabbani and Ahn, 2019 ▶; Wei et al., 2009 ▶) as well as the protective effects of natural compounds (Khan et al., 2020 ▶).

Previous studies have shown that *T. terrestris* has antiproliferative activity in tumor cell lines, characterizing the antitumor potential (Divya et al., 2014 ▶; Pourali et al., 2017 ▶). In addition to these data, it is known that selective cytotoxicity is a desired characteristic of a new antitumor compound (Abraham and Staffurth, 2020 ▶). A special group of saponins stand out among the active antitumor compounds, as they can act in a way similar to steroid hormones, producing an effect on hormone-sensitive organs, positively interfering with their homeostasis, acting as chemoprotective substances against various types of cancer, including lung, breast, glioma, colon, leukemia and prostate cancer. These saponins have antiproliferative, anti-invasive, antiangiogenic, antimetastatic effects and may induce apoptosis and/or necrosis (Kim et al., 2011 ▶; Zhao et al., 2018 ▶).

*In vitro* antiproliferative activity of *T. terrestris* extracts was examined in human tumor cell lines using doxorubicin as the standard. The saponins-enriched extract showed higher selectivity for the kidney cell lines and the standard extract presented moderate activity against tested cell lines. Siva et al. (2017) ▶ also used doxorubicin as a reference drug in tumor cell lines in comparison to *Cipadessa baccifera *extract and observed similar results. Other results found by Shamah-Levy et al. (2006) ▶, demonstrated that *T. terrestris* showed toxicity but it could be used for the treatment of various diseases. However, extracts were able to induce more than 50% of cell lines death at concentrations of 0.25-250 µg/ml.

The implications in production, signaling and physiological concentration of AGEs are mutually involved in the cancer genesis and complications of oxidative stress and in the promotion and/or aggravation of degenerative diseases such as cancer and diabetes. This suggests that a multi-directed therapeutic approach is imperative to control these consequences (Anis and Sreerama, 2020 ▶). As already discussed in different studies, multiple activities and different molecular pathways are blocked by steroidal saponins (Wang et al., 2019 ▶). This molecule is able to completely block RAGE (Receptor for Advanced Glycation End-products) signaling pathways (Chhipa et al., 2019 ▶). Thus, interventions including this molecule may be beneficial in controlling and preventing diseases promoted by glycation agents and exert benefits in the control of cancer cell lines growth and proliferation.

According to the results, it was possible to conclude that standardized and saponins-enriched extracts of *T. terrestris* present antiglycation and antioxidant activity, and antiproliferative potential in human-tumor cells lines. However, the extract enriched with saponins showed a greater activity in the evaluations performed, demonstrating that the steroidal saponins present in the herbal extract may be directly associated with the activities investigated in the present study.
